# Mapping insoluble indole metabolites in the gastrointestinal environment of a murine colorectal cancer model using desorption/ionisation on porous silicon imaging

**DOI:** 10.1038/s41598-019-48533-2

**Published:** 2019-08-26

**Authors:** David Andre Rudd, Kirsten Benkendorff, Charndeep Chahal, Taryn Guinan, Ove Johan Ragnar Gustafsson, Babak Esmaeelian, Hanna Krysinska, Lisa Pogson, Nicolas Hans Voelcker, Catherine Anne Abbott

**Affiliations:** 1 Marine, Ecology Research Centre, School of Environment, Science and Engineering, Southern Cross University, Lismore, NSW 2480 Australia; 2 Melbourne Centre for Nanofabrication, Victorian Node of the Australian National Fabrication Facility, Clayton, VIC 3168 Australia; 3 Drug Delivery, Disposition & Dynamics, Monash Institute of Pharmaceutical Sciences, Monash University, Parkville, VIC, 3052 Australia; 4 College of Science and Engineering, Flinders University, Bedford Park, SA 5042 Australia; 5 ARC Centre of Excellence in Convergent Bio-Nano Science & Technology, Future Industries Institute, University of South Australia, Mawson Lakes, SA 5095 Australia; 6 Flinders Cancer Research, Flinders University, Bedford Park, SA 5042 Australia; 7 Commonwealth Scientific and Industrial Research Organisation (CSIRO), Clayton, VIC 3168 Australia; 8 Monash University, Clayton, VIC 3168 Australia

**Keywords:** Two-dimensional materials, Imaging studies, Natural products, Cancer models

## Abstract

Indole derivatives are a structurally diverse group of compounds found in food, toxins, medicines, and produced by commensal microbiota. On contact with acidic stomach conditions, indoles undergo condensation to generate metabolites that vary in solubility, activity and toxicity as they move through the gut. Here, using halogenated ions, we map promising chemo-preventative indoles, i) 6-bromoisatin (6Br), ii) the mixed indole natural extract (NE) 6Br is found in, and iii) the highly insoluble metabolites formed *in vivo* using desorption/ionisation on porous silicon-mass spectrometry imaging (DIOS-MSI). The functionalised porous silicon architecture allowed insoluble metabolites to be detected that would otherwise evade most analytical platforms, providing direct evidence for identifying the therapeutic component, 6Br, from the mixed indole NE. As a therapeutic lead, 0.025 mg/g 6Br acts as a chemo-preventative compound in a 12 week genotoxic mouse model; at this dose 6Br significantly reduces epithelial cell proliferation, tumour precursors (aberrant crypt foci; ACF); and tumour numbers while having minimal effects on liver, blood biochemistry and weight parameters compared to controls. The same could not be said for the NE where 6Br originates, which significantly increased liver damage markers. DIOS-MSI revealed a large range of previously unknown insoluble metabolites that could contribute to reduced efficacy and increased toxicity.

## Introduction

The ability of mass spectrometry imaging (MSI) to spatially discriminate a drug from its metabolites, against the complex background of endogenous compounds, has enabled it to emerge as a revolutionary technology in pharmaceutical research^1–3^. However, the sensitive detection of low mass ions using matrix-assisted laser desorption ionisation-MSI (MALDI-MSI) is hampered by matrix-specific background ions in the low mass range^4,5^. This has driven the emergence of matrix free alternatives, including nanostructure imaging mass spectrometry (NIMS)^6^. One NIMS variant, desorption ionisation on porous silicon (DIOS), relies on an electrochemically etched porous silicon (pSi) substrate with a high surface area (up to 800 m^2^/g) and a high UV cross-section^7^. The advantages of DIOS for imaging complex tissue include improved signal intensity^5^, reduced background spectral noise^8,9^, high efficiency desorption of ions and sensitivity down to yoctomole quantities^10^. The key advantage of DIOS, as compared to other drug metabolite discovery methods, is the capacity to detect and map metabolites that are not amenable to other pharmacokinetic approaches^11^. DIOS-MSI can map spatially-preserved tissue distributions for metabolites which only solubilise in solvents that are unsuitable for traditional pharmacology.

Low concentrations of drug metabolites in complex environments, like the gastrointestinal (GI) lumen, are often masked by primary metabolites, reducing confidence when annotating spectra obtained from *in situ* analyses^12^. Bromine – which naturally exists as two stable isotopes (^79^Br 50.69% and ^81^Br 49.31%) – provides a distinctive mass tracer that can be identified within the highly complex low molecular weight range of a mass spectrum^13^. Brominated structures are, therefore, ideally suited to tracking low abundant drug metabolites.

Indole derivatives are a class of bioactive compounds for which drug absorption, distribution, metabolism and excretion (ADME) is not easily predicted. Indole metabolites can form in the GI environment from bacterial catabolism of dietary tryptophan and are present as part of diet microbiome interactions, as well as from administered drugs^14,15^. Indoles are the structural base for a number of drugs and plant constituents that are used, or recommended, in the treatment and prevention of a range of cancers^16–18^. Recent research has also shown them to extend life span in animal models, reducing age related disease burden to improve health into later age^19^. However, a number of indole derivatives demonstrate dichotomous effects in tumourigenesis, potentially acting as ligands for a range of tumour associated targets^20^. Therefore, there is a demonstrable need to identify the range of metabolites produced *in vivo* for indole-based constituents.

Monomeric and dimeric brominated indoles from Muricidae molluscs have shown a broad range of pharmacologically active properties^18,21,22^, several of which have led to unmodified and structurally modified patented drugs^23–26^. The most notable of these are the indirubins, which are potent modulators of glycogen synthase kinase-3 (GSK-3)^27^, cyclin-dependent protein kinases (CDKs)^28,29^ and ligands for the aryl hydrocarbon receptor^20,24^. Both *in vitro*^30^ and *in vivo*^31^ experiments have been undertaken with this group of compounds to establish their chemo-therapeutic applications^32,33^. Although crucial, very few studies have determined the active structure *in situ* at the *in vivo* sites of activity.

**Figure 1:**
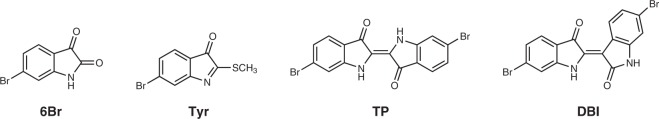
Mono and di-brominated indoles found in NE including the chemo-preventative candidate 6-bromoisatin (6Br; m/z 224.9, 226.9), tyrindoleninone (Tyr; m/z 254.9, 256.9), 6,6′-dibromoindigo (Tyrian purple, TP; m/z 417.9, 419.9, 421.9) and 6,6′-dibromoindirubin (DBI; m/z 417.9, 419.9, 421.9).

The monomeric basis of indirubin, 6-bromoisatin (6Br), and the marine mollusc extract from which it is obtained, effectively induces apoptosis in early colon cancer models^34,35^, but the mechanism behind these effects is unknown. In this study we have used a long term genotoxic carcinogen (azoxymethane, AOM) animal model to further examine the ability of these compounds to protect against colon cancer. AOM induces the development of tumours that share many of the histopathological characteristics of human colorectal cancer, so correlations between histopathology and drug metabolites are relevant to some human CRC processes^36^. Herein, we have demonstrated the *in situ* tracking of 6Br (Fig. 1), a promising chemo-preventative compound, and the metabolites in the marine extract it originates from (NE; Fig. 1, Supplementary Fig. S1), in the context of the compound’s therapeutic activity. This approach will provide further insight into the complex effects of the GI tract on orally administered indole derivatives, in the form of isolated drug candidates and complex mixtures, and has the potential to establish the dichotomous therapeutic and toxic effects of indole metabolites^20^.

## Results and Discussion

### DIOS as an analytical platform for analysing absorption, distribution, metabolism and excretion of brominated xenobiotic compounds

**Figure 2:**
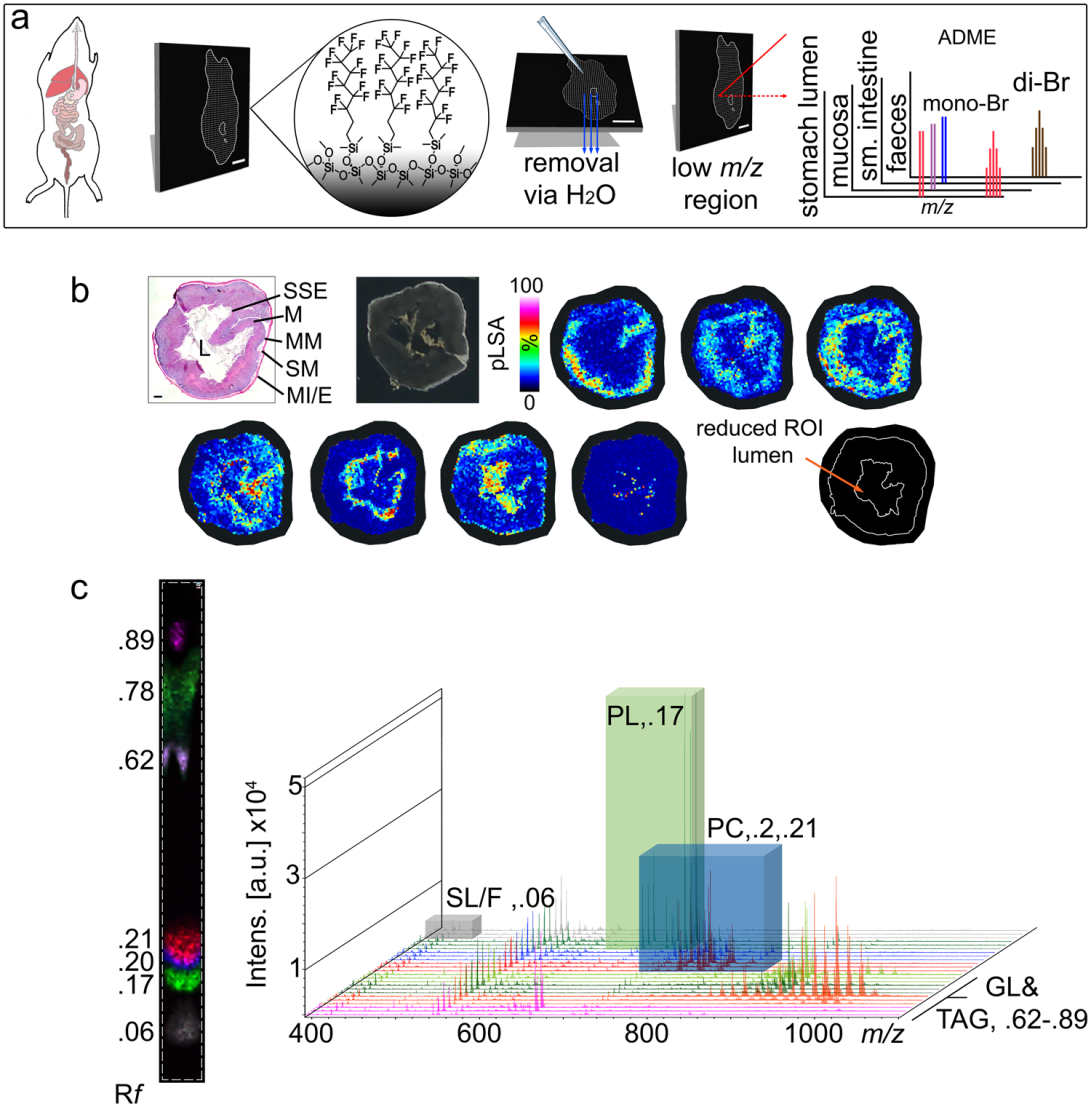
DIOS-MSI of the GI tract and the applied chemo-informatic approach to metabolite discovery. (**a**) Workflow (left to right) from colorectal mouse model to DIOS-MSI mapping. (**b**) pLSA of total spectral data of >3,000 to create regions of interest for analysis. H & E section: lumen (L), stratified squamous epithelium (SSE), mucosa (M), muscularis mucosae (MM), sub-mucosa (SM) and muscularis interna/externa (MI/E). Scale bar 2 mm. (**c**) Comparative lipid TLC-MALDI imaging data of remaining tissue for peak annotation and de-replication of lipid signatures from brominated metabolites, with corresponding R*f* values for lipid class TLC spots; labelling: SL/F, short chain lipids, fatty acids or fragments; PL, phospholipids; PC, phosphocholines; GL, unannotated glycerolipids; TG, triacylglycerides.

To investigate the *in vivo* metabolism of 6Br and NE (Fig. 1) mice were treated by daily oral gavage for 14 weeks with oil, 6Br or NE in a colon cancer prevention model (Supplementary Fig. S1). Treatment was delivered 4 h prior to the mice being sacrificed and GI tissue subjected to metabolomic spatial profiling using DIOS-MSI (Fig. 2a). We detected a range of monomeric and dimeric brominated indoles in the GI tissue of treated mice (see Supporting Information), which were not detected in control mice. The brominated metabolites were readily identified by their traceable isotopic peak patterns (Fig. 2, Supplementary Fig. S2), even though the *m/z* fall below the typical point at which matrix ions interfere with MALDI-MS (*m/z* < 700).

In our model, brominated indole metabolites appeared together with a considerable amount of non-target primary metabolites in the same spectral region (Fig. 2), typically producing >3,000 spectra per section. Reducing this data to a manageable subset is essential when searching for all possible drug metabolites, including insoluble ones. Smaller regions of interest (ROI) can be identified by a statistical pipeline, probabilistic latent semantic analysis (pLSA), using direct comparison to tissue regions in the serial haematoxylin and eosin (H & E) section^37^. Selected ROI can then be screened for brominated signatures (Fig. 2b, Supplementary Fig. S3).

Endogenous ions were validated by solvent phase-extraction of lipids from the remaining stomach tissue and contents, followed by thin layer chromatography (TLC) coupled to the MALDI MS (TLC-MALDI, Fig. 1c). This approach was used to add chromatographic separation using a TLC plate, allowing fragmentation to annotate lipids associated with the primary cell components of tissue from the different regions (Fig. 2c, Supplementary Figs S4 and S5). The profiling of non-target lipids is important for separating out di-brominated signatures from lipids in the *m/z* 400 to 1100 range, where isotopic patterns of lower abundant lipids can appear similar to a typical di-brominated spectral pattern. Comprehensive identification of the lipophilic fraction of the tissue extract is not entirely necessary, but being able to verify whether a putative signal is related to a drug metabolite is essential. TLC-MALDI provides a means for rapidly acquiring and identifying lipid class data without onerous analytical purification (Supplementary Figs S4 and S5).

### *In vivo* efficacy of 6Br and *in situ* metabolites detected by DIOS MSI

**Figure 3:**
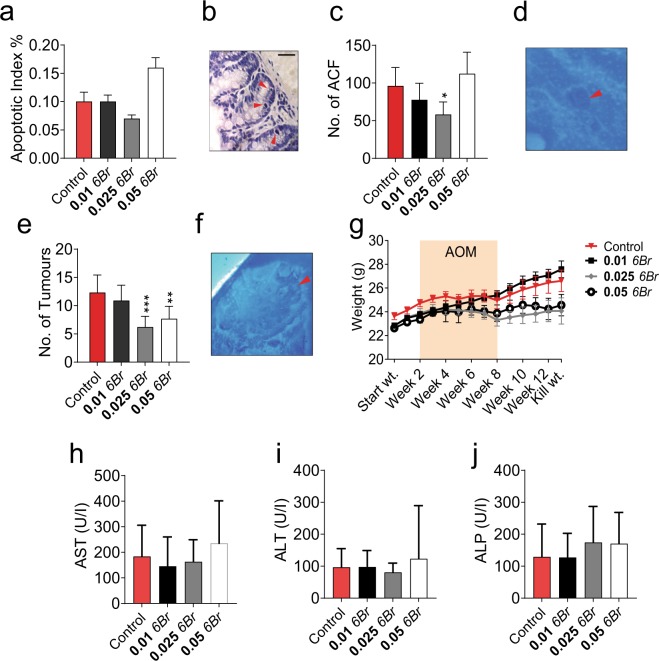
Therapeutic effect of 6Br (mg/g) and effects on liver function markers after 14 weeks of daily oral gavage and 6 weekly intraperitoneal injections of 10 mg/kg azoxymethane in an early stage colorectal cancer prevention model. (**a**) Apoptotic index in colon crypts and (**b**) representative haematoxylin stained colon section showing apoptotic cells (red arrows), scale bar 50 µm. (**c**) Aberrant crypt foci (ACF) count in excised colons and (**d**) representative ACF. (**e**) Tumour count and (**f**) representative tumour. Colon epithelium images stained with 0.2% methylene blue showing a tumour or ACF (red arrow) (**g**) Mouse weight. Levels of liver enzymes in the blood of mice, (**h**) aspartate aminotransferase (AST), (**i**) alanine aminotransferase (ALT) and (**j**) alkaline phosphatase (ALP). All treatment groups are compared to control mice by ANOVA: P ≤ 0.05 (*), P ≤ 0.01 (**) and P ≤ 0.005 (***). Values represent means and error bars ± standard deviation (SD).

To investigate the *in vivo* efficacy of 6Br for preventing aberrant crypt foci (ACF) formation, mice were continuously treated with different doses of 6Br for 14 weeks. After the first two weeks of treatment, animals received six weekly injections of the genotoxic carcinogen, AOM, at 10 mg/kg^38^. Oral treatment with 6Br significantly reduced the development of ACF from damaged colon mucosa in the 0.025 mg/g group (*P* ≤ 0.01; Fig. 3d) but not at the 0.05 mg/g higher dose. More importantly, tumour numbers were significantly reduced in the colon of treated mice compared to the controls (analysis of variance [ANOVA] *P* < 0.0001), for both 0.025 mg/g (*P* ≤ 0.001) and 0.05 mg/g (*P* ≤ 0.01) 6Br treatment groups (Fig. 3e). In addition, the proliferation index was also significantly decreased in colons of treated mice compared to controls, for 0.01 mg/g 6Br (P ≤ 0.005) and 0.025 mg/g 6Br (P ≤ 0.0001) treatment groups but not at the highest dose 0.05 mg/g (Supplementary Fig. 7a). The apoptotic index increased in the 0.05 mg/g group (*P* ≤ 0.05; Fig. 3a) but not at lower doses. The protective effects of 6Br against ACF and subsequent tumour formation in this study do not appear to be dose dependent and results mainly from a decrease in proliferation rather than from another mechanism. Previous *in vitro* studies showed that 6Br reduces the viability of HT-29 colorectal adenocarcinoma cells in a dose dependent way between 0.025 and 0.1 mg/g^35^. In addition utilising a short term *in vivo* acute-apoptotic response to a genotoxic carcinogen (AARGC) mouse model, 6Br at the higher doses (0.05 and 0.1 mg/g) significantly increased the apoptotic index and reduced proliferation in distal colon crypts six hours after a single 15 mg/g dose of AOM^35,39^. These early response protective effects were not observed at the lower 0.025 mg/g 6Br dose^35^. However, this latest study expands on the previous experiments by demonstrating that six weeks after a longer regime of carcinogen exposure, 0.025 mg/g 6Br treatment is able to reduce ACF number and tumour formation. Further studies are required to fully understand the mechanism behind this protection.

**Figure 4:**
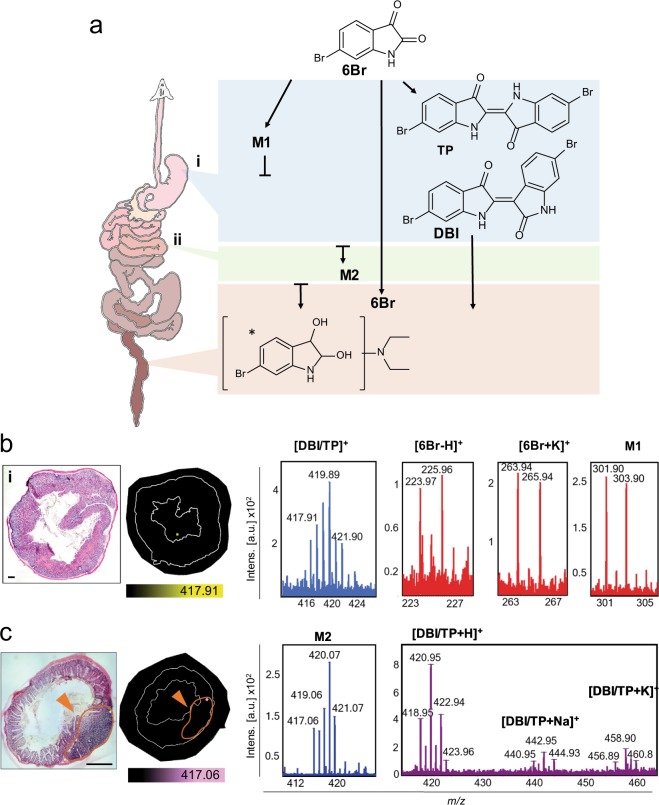
DIOS-MS and -MSI detection of 6Br in the GI tract of a colorectal cancer murine model, at therapeutic 6Br concentrations. (**a**) Schematic of GI tract and sampling locations for imaging (i, ii) for the corresponding DIOS-MSI analyses (**b,c**), as well as putative structures of 6Br metabolites. *Putative structure based on ESI-UHR-QqTOF calculations (Supplementary Fig. S10). (**b**) H & E section, DIOS-MSI map of the stomach with corresponding brominated spectra. (**c**) H & E section, DIOS-MSI map of the ileum with corresponding brominated spectra. Orange arrows indicate gut associated lymphoid tissue. Scale bars 2 mm.

MSI was used to highlight the GI distribution of 6Br and its metabolites after oral delivery to mice (Fig. 4). DIOS-MSI confirmed 6Br in the GI using Br ion pairs, *m/z* 223.977, 225.965 [M-H]^+^ and 263.945, 265.946 [M + K]^+^ (C_8_H_4_BrNO_2_) (Fig. 4b). The former ion is generated by hydride abstraction from the indole structure, previously observed during MSI analysis of 6Br and is seen when using ESI-MS to detect other indoles^11,40^. Additionally, a brominated metabolite (M1) (*m/z* 301.902 and 303.906) and a dimer with a dibrominated isotopic profile (centered on *m/z* 419.891) were detected in the acidic murine fore-stomach (Fig. 4a,b). These signals were confirmed in frozen homogenates of experimental tissue and control tissue sections injected with synthetic 6Br and TP after tissue collection (C_16_H_8_Br_2_N_2_O_2_) for comparison to non-impregnated controls (Fig. 2a; Supplementary Fig. S6). The major acid condensation dimer of 6Br, *m/z* 418.891, was not detectable by liquid chromatography mass spectrometry (LC-MS), high resolution MS or gas chromatography MS (GC-MS; Supplementary Fig. S8), even after extraction in a range of different solvents, because they failed to solubilise the major metabolites (methanol, chloroform, *n*-hexane, dimethyl sulfoxide (DMSO) and dimethyl formamide (DMF)). A Log *p* of 4.47 (octanol: water partition coefficient model) makes DBI/TP insoluble in many solvents^41,42^. The detection of this dimer by DIOS-MSI in the low pH stomach environment, in combination with its known insolubility, suggests that some DBI/TP passes through the GI unmodified.

The *in situ* metabolism of bioactive compounds has the potential to generate metabolites that may cause known side effects or unpredictable off-target effects. Our 14 week mouse model, indicates there is no significant toxicity associated with the oral administration or *in vivo* metabolism of 6Br. There was no significant difference in body weight when comparing 6Br treated mice to controls (ANOVA *P* ≤ 0.3752; Fig. 3g), with all mice gaining weight over the entire study period and appearing generally healthy during the post AOM injection period. Furthermore, we found no significant differences in liver damage markers aspartate aminotransferase (AST, *P* = 0.6011; Fig. 3h), alanine aminotransferase (ALT, *P* = 0.7918; Fig. 3i), and alkaline phosphatase (ALP, *P* = 0.6156; Fig. 3j). Similarly, in previous studies, mice orally administered synthetic 6Br, or 6Br purified from the NE, for two to four weeks did not exhibit any apparent hepatotoxicity based on plasma levels of liver enzyme damage markers AST, ALT and ALP^35,39^. However, 6Br was found to have some possible immune-modulatory effects, causing a decrease in white blood cell counts in one study^35^ and an increase in another^39^. In this study, where 6Br was administered for 14 weeks, it had immune-modulatory effects compared to controls, with the highest 6Br group (0.05 mg/g) causing a significant increase in the white blood cell count and an increase in lymphocytes (Supplementary Fig. S9). Inflammatory responses, particularly those seen during chronic inflammatory conditions, can affect most of the stages of tumourigenesis including initiation, promotion, malignant conversion, invasion and metastasis^43^.

Interestingly, DIOS-MSI revealed an additional metabolite, M2 in mice treated with 6Br, which was detected in the lower ileum (*m/*z 419.062) situated in enlarged mucosa-associated lymphoid tissue (Fig. 4c, orange arrows). The location of this dibrominated ion within a lymph node suggests that some form of lymphatic system interaction may occur, which could be attributable to either the lipophilic nature of the structure itself or the incorporation of 6Br in oil for oral delivery in our animal model. Lipid–based formulas have been shown to facilitate uptake into the lymphatic system to avoid first pass metabolism for compounds with low bioavailability^44,45^. The *in situ* detection of a brominated metabolite in lymphoid tissue is an interesting finding, as the lymphatic system is increasingly becoming a target for the treatment of chronic inflammation due to its role in immune surveillance of the lumen environment^43^. 6Br has been shown to significantly inhibit pro-inflammatory mediator signalling pathways and prevent translocation of nuclear factor kappa B (NFκB) into the nucleus of lipopolysaccharide (LPS) stimulated macrophages *in vitro*^21^. It has also been shown to successfully ameliorate the early inflammatory response and protect lung architecture in a mouse model for acute lung inflammation^22^. Future application of DIOS-MS in other disease models for inflammation and cancer has the potential to track the uptake of 6Br and its metabolites using different modes of delivery to more clearly elucidate potential multi-modal action for its chemo-preventative activity.

To confirm which of the brominated metabolites were present in the colonic environment from our 14 week mouse model for colon cancer, faecal matter was collected and spotted onto DIOS surfaces and the remaining material extracted by phase partition with chloroform: methanol for ESI-ultra high resolution-quadrupole time-of-flight (ESI-UHR-QqTOF) MS. ESI-UHR-QqTOF of faecal extracts confirmed that 6Br is also present in the colonic lumen, detected at *m/z* 223.9345, 225.9324 [M-H]^+^ (Supplementary Fig. S10). An additional brominated signature was observed in faecal extracts by ESI-UHR-QqTOF, M3 *m/z* 301.0549, 303.0529, (predicted as C_12_H_18_BrN_2_O_2_) and assuming this compound has the indole core structure, it would correspond to the diethylamino derivative of 6Br (M3; Fig. 4a and Supplementary Fig. S10). The dimers detected by DIOS were undetectable using ESI-UHR-QqTOF.

Based on our DIOS-MSI results, it is uncertain whether 6Br is directly exerting an apoptotic and tumour reducing effect or indirectly acting via metabolites that form in the GI. The other 6Br metabolites detected, M1 – M3 (Fig. 4a), could be imparting a therapeutic effect in what is a highly complex metabolic environment. The power of DIOS-MSI to detect low abundant metabolites produced *in vivo* is highly advantageous, as some would typically evade traditional pharmacological approaches in ADME studies. Chemical synthesis of the diethylamino metabolite of 6Br with N or O substitution, would enable comparison to the colonic metabolite of 6Br, M3, both to confirm the structure and test whether this compound can promote an apoptotic response. N-substituted indoles have previously shown promise as chemo-preventative agents^46,47^.

### *In vivo* activity and *in situ* metabolism of the nature extract (NE)

**Figure 5:**
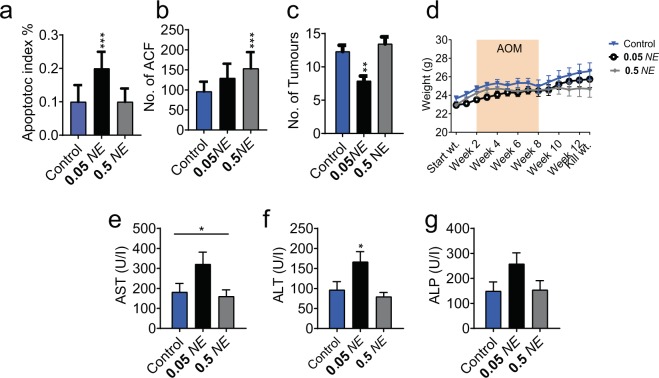
Therapeutic effect of *Dicathais orbita* hypobranchial gland extract (NE, mg/g) and effects on liver function markers after 14 weeks of daily oral gavage and 6 weekly intraperitoneal injections of 10 mg/kg azoxymethane in an early stage colorectal cancer prevention model. (**a**) Apoptotic index in colon crypts, (**b**) aberrant crypt foci (ACF) and (**c**) tumour count in excised colons per mouse, (**d**) mouse weight, (**h**) aspartate aminotransferase (AST), (**i**) alanine aminotransferase (ALT) and (**j)** alkaline phosphatase (ALP) levels in the blood of mice. All treatment groups are compared to control mice by ANOVA: p ≤ 0.05 (*), p ≤ 0.01 (**) and p ≤ 0.005 (***). Values represent means and error bars ± SD.

Consistent with our previous genotoxic carcinogen models which studied apoptosis six hours after a single 15 mg/kg AOM dose^34,39^ the lower 0.05 mg/g NE treatment significantly increased apoptosis and significantly decreased proliferation in epithelial cells of colon crypts (Fig. 5a, Supplementary Fig. 7d). Whilst this lower 0.05 mg/g NE dose did not lead to a significant reduction in ACF (Fig. 5b), anti-cancer effects were observed as there was a significant reduction of tumours in the colon of mice on this treatment (Fig. 5c). In contrast the higher NE dosage (0.5 mg/g) which contained a similar concentration of 6Br to the 0.025 mg/g synthetic 6Br treatments, gave similar results to 0.025 mg/g 6 Br and was able to significantly reduce colon cell proliferation, but was unable to induce apoptosis (Fig. 5a, Supplementary Fig. 7d). Unexpectedly, the higher NE dose led to a significant increase in ACF number, despite the observed decrease in proliferating cells but this increase in ACF did not lead to a significant increase in tumour numbers (Fig. 5b,c). Interestingly, the lower 0.05 mg/g NE dose showed more anti-cancer activity than 0.01 mg/g synthetic 6Br, despite having a lower predicted concentration of 6Br as calculated in Valles-Regino *et al*.^48^ (~0.0044 mg/g). This suggests that other metabolites in the extract could also be involved in this anti-cancer activity at lower concentrations or may be converting directly to 6Br. The NE had no effects on mouse body weight (Fig. 5d). The inability of the higher dose of NE to provide anti-cancer effects relative to the 10-fold lower concentration was unexpected, but is consistent with the U shaped hormetic response previously reported for this extract^49^. It is likely that complex feedback cycles may regulate the detoxification pathways and clearance rates of the bioactive indoles *in vivo*. These surprising results underscore the importance of elucidating the contribution of individual components in natural extracts for efficacy testing.

**Figure 6:**
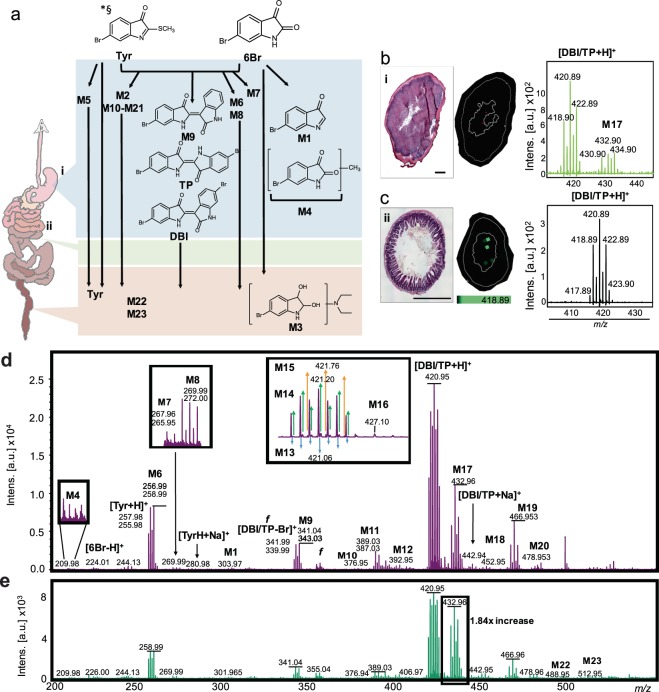
DIOS-MSI maps the NE in the GI tract of a murine colorectal cancer model. (**a**) Putative structures of NE metabolites detected in the GI and a schematic of the GI tract, showing sampling locations (i, ii) for corresponding DIOS-MSI analysis. Symbols: *ESI-UHR-QqTOF calculated structure; and § ion detected in LC or GC-MS. H & E section, DIOS-MSI maps and corresponding spectra of the major di-brominated metabolites detected, (**b**) in stomach, and (**c**) ileum sections *in situ*. Scale bar 2 mm. (**d**) Raw spectra from 0.5 mg/g NE stomach tissue showing most of the major brominated ions, M4 – M23 (with zoomed view of minor ion and the DBI/TP region), and (**e**) comparative raw spectra from faecal extract showing the a relative increase in the proportion of M17.

To demonstrate the power of DIOS-MSI for tracking the *in vivo* metabolism of more complex mixtures, we also tracked the fate of NE in the GI tract of mice from the 14 week colon cancer prevention model (Fig. 6a–c; Supplementary Fig. S2d). After oral administration of the NE, 6Br was detected as the hydride abstraction [M-H]^+^ and [M + K]^+^ adduct (Fig. 6d). Consistent with previous analyses of brominated compounds in mollusc extracts Valles-Regino *et al*.^48^ (Supplementary Fig. 2d), those used for oral gavage, showed the methane thiol containing brominated indole derivative Try was detected at *m/*z 255.982, 257.983 ([M + H]^+^) and 278.990, 280.982 ([MH + Na]^+^, C_9_H_6_BrNOS). An additional seven monomeric brominated signatures were detected in the stomach of NE treated mice (Fig. 6), none of which were detected in the control mice. This diversity of brominated compounds is likely the result of the gastric environment causing acid condensation, hydrolysis and potential substitution of reactive functional groups. The natural dimers, TP or DBI, were also detected as protonated ([M + H]^+^) and sodiated ([M + Na]^+^) adducts (Fig. 6b,d). The *m/z* for dibromoindigo (418.89, 420.89, 422.89) was validated against a TP standard homogenised in control stomach (Supplementary Fig. S2e). Additionally, 11 dimeric brominated signatures, with varying ion abundance, were detected in the stomach tissue (Fig. 6d). All brominated signatures detected by DIOS-MSI from the stomach were distributed in the stomach lumen (Fig. 6) and, interestingly, were detected up to 4 h post gavage. Mice consume chow in small successive quantities. Hence, gastric emptying is much slower relative to humans and stomach contents are thus progressively diluted over time^50^.

In comparison to *in situ* detection using DIOS-MS, conventional extraction and analysis techniques were unable to detect the same complexity of brominated metabolites. Only the two major brominated indoles in the NE could be detected by GC-MS of stomach extracts (Supplementary Fig. S8): these were 6Br at *m/z* 224, 226, a methylated metabolite of 6Br at *m/z* 239, 241 ([M + CH_3_]^•+^) and Tyr at *m/z* 255, 257 ([M]^•+^). A single dimer was detected *via* LC-MS (Supplementary Fig. S8) which was also present in DIOS mass spectra, at *m/z* 474.9, 476.9, 478.9, with a fragment ion pair at *m/z* 247.9 and 249.9. Using DIOS-MSI, the ileum and digesta were found to contain the same complexity of monomeric and dimeric brominated metabolites detected in the stomach, except M6 (*m/z* 265.95 and 267.96), and contained an additional monomer pair at *m/z* 488.955 and 490.967 (M22), as well as a dimer centred on *m/z* 512.956 (M23, Fig. 6e). In extracts from faeces collected during excision of the colon, ESI-UHR-QqTOF was only able to detect 6Br and Tyr compared to DIOS-MS, and an additional eight monomer Br adduct ions, but no dimers (Supplementary Fig. S10). DIOS-MS is therefore ideally suited for the detection of poorly soluble metabolites in faeces, as well highlighting the biodistribution of complex metabolites in the GI.

One of the major differences observed between the brominated profile of the stomach, as compared to the lower ileum and digesta, was a 1.84 fold relative increase in the dimer signature centred on *m/z* 432.963 (M17, Fig. 6e), a mass difference of 13 relative to DBI/TP. This mass difference is typically seen during methylation of dibrominated indole/isatin structures as they pass through the GI tract, which could come from xenobiotic detoxification reactions in the liver. The AOM rodent model of colon cancer relies on P450 hepatic and GI processing of the genotoxic procarcinogen AOM to the carcinogen methylazoxymethanol (MAM), which subsequently acts as a methylating agent in the formation of alkylated DNA adducts^51^. The hepatic GI processing of AOM to MAM is carried out by cytochrome P450s, primarily CYP2E1^52,53^. Brominated indole metabolites reaching the liver, or P450 sites in the GI tract, could specifically compete for the CYP450 binding site with AOM. Competition for AOM metabolism would prolong the presence of MAM in the colonic environment. This could counteract the therapeutic value of the brominated indoles at the time of carcinogen delivery and may explain why mice treated with the higher dose of NE showed a significant increase in ACFs and no reduction in tumours (Fig. 5). Further work on the differences in the xenobiotic CYP450 metabolism between 6Br and NE metabolites would prove interesting if competition with AOM metabolism is occurring^54^.

Some of the brominated indoles in the NE or the metabolites generated *in vivo* do appear to be more toxic than 6Br. Significant non-dose dependent effects of NE were found on the liver enzyme AST, with higher overall AST concentrations found in the blood serum of mice in the 0.05 mg/g treatment groups compared to controls (ANOVA *P* = 0.0453; Fig. 5e). Consistent with this, Esmaeelian *et al*. reported significantly elevated levels of AST in mice treated with Tyr for only two weeks before the injection of the AOM carcinogen in the acute apoptotic response to genotoxic carcinogens animal model^39^. Furthermore, we detected significantly elevated ALT concentrations in the mice treated with 0.05 mg/g NE compared to controls (*P* = 0.0108; Fig. 5f). These effects on liver enzymes were not seen at the higher dose of 0.5 mg/g, suggesting the higher dose may be detected and metabolised differently *in vivo*. NE had no effect on ALP when administered for 2 weeks in the acute apoptotic response to genotoxic carcinogens animal model^39^ or when administered for 14 weeks in this longer-term cancer model (*P* = 0.1152; Fig. 5g). Nevertheless, the increased AST and ALT at the lower NE dose supports the idea that some of the other brominated indoles in the NE, or their metabolised by-products, may have some off-target side effects that lead to liver damage. Additionally, the immunomodulatory effects of the NE where different to those observed with 6Br. The NE extract had no significant effect on red blood cells or lymphocytes however 0.05 mg/g NE (*P* = 0.0231; Supplementary Fig. S11) led to a significant increase in monocyte number.

Overall our *in vivo* model demonstrates the efficacy of 6Br and the NE, but with interesting dose effects for both the 6Br and NE justifying further investigation of the metabolic pathways involved. DIOS-MSI reveals a diversity of brominated metabolites formed in the GI tract from orally administered NE (Fig. 6), providing metabolite data that could explain the therapeutic variations seen in mice treated with NE compared to 6Br alone.

## Conclusion

DIOS-MSI effectively captured brominated metabolites of 6Br and the more complex NE, allowing further investigation of the major metabolites at the site of therapeutic action. 6Br was found to be an effective chemo-preventative compound when orally administered in a 14 week colorectal cancer model. DIOS-MSI results showed that 6Br passes through the digestive tract, with relatively few metabolites forming *in vivo* and causing negligible deleterious side effects on blood biochemistry and general health parameters. The bioavailability of 6Br in the colon as seen by means of DIOS-MSI correlates with the observed protective effects, inducing apoptosis in damaged epithelial cells and preventing the progression from ACF to tumour formation. The same conclusion could not be attributed to the NE from which 6Br originates, either as a consequence of a considerable increase in metabolite species (which were demonstrated to form *in situ* by DIOS-MSI), or due to the impact of specific metabolites on the metabolism of AOM in the GI tract or liver.

Overall, MSI provides an effective tool for establishing the ADME properties of natural extracts, purified compounds and dietary constituents: a process simplified by bromination, which produces a characteristic isotopic pattern for easy detection. The versatility of DIOS, and the potential to alter the functionalisation of the surfaces, particularly when combined with high mass resolution, is likely to have a considerable impact on mapping low molecular weight constituents in tissues.

## Experimental

### Materials

All solvents used were chromatography grade. Methanol, hydrofluoric acid (HF, 48%^5^), and chloroform were obtained from Merck (VIC, Australia). Ethanol was purchased from Chem Supply (SA, Australia). Tridecafluoro-1,1,2,2-tetrahydrooctyl)dimethylchlorosilane (F_13_) was purchased from Gelest Inc. (PA, USA). 6Br was synthesised by Tokyo Chemical Industry (Tokyo, Japan), the structure and purity were validated according to Esmaeelian, *et al*.^35^. The natural extract (NE) was prepared from the hypobranchial glands of the marine gastropod *Dicathais orbita* according to Esmaeelian, *et al*.^55^.

### *In vivo* murine model

Male C57BL/6 mice at 10 weeks of age were purchased from the Animal Research Centre (Perth, Australia). During experimental conditions mice were kept in groups of four per cage, given food (standard rodent chow) and water *ad libitum*, and maintained at 22 ± 2 °C on a 12 h light/dark cycle. To assess the effects of 6Br and NE on general health parameters mice were weighed weekly, or daily if showing signs of weight loss, and monitored daily for signs of ill health. A minimum of 8 replicate mice were used in each treatment and control group. As AOM has a significant impact on the liver^56^, mice showing a continual weight loss of more than (10%), continual signs of ill health or significant deviations from expected ethics parameters (3 continuous days), were removed from the study. All animal work was conducted according to the Australian Code of Practice for the Care and Use of Animals for Scientific Purposes under the Flinders University Animal Ethics Committee approval notice 751-10.

The experimental design was based on an established ACF model for the early stage prevention of colorectal cancer^57^, however genotoxic damage was induced by six weekly intraperitoneal injections of 10 mg/kg AOM, instead of the previously described four doses of 15 mg/kg. As 6Br is a lipophilic compound, mice were administered the desired concentration dissolved in 100 μL of sunflower oil with 0.02% vitamin E via oral gavage. Mice were randomly assigned to an oil control group (*n*** = **11); 6Br at 0.05 mg/g (*n* = 8), 0.025 mg/g (*n* = 11) and 0.01 mg/g (*n* = 12), or NE at 0.5 mg/g (*n* = 11) and 0.1 mg/g (*n* = 11). Oral gavage of treatments, or oil control, was administered daily for 14 weeks and 4 h prior to the mice being killed. The first AOM dose was delivered two weeks after oral gavage of treatments began.

Mice were sacrificed using a single injection of ketamine (75 mg/kg) and xylazine (5 mg/kg), followed by cardiac bleed immediately prior to cervical dislocation. Blood was collected into sodium heparinised-EDTA vials (BD Microtainer®) and sent to a pathology clinic (Gribbles Pathology, South Australia, Australia) to test blood biochemistry and haematology parameters. The samples were run through the Abbott Cell Dyn 3700 analyzer for haematology assessment and Siemens Advia 1800 chemistry analyzer for biochemistry analysis. The blood levels of liver enzymes including aspartate aminotransferase (AST), alanine aminotransferase (ALT), and alkaline phosphatase (ALP) were assessed as indicators of hepatotoxicity.

Mice were dissected immediately post kill, after which colons were collected to evaluate signs of early stage cancer formation^38^ whilst stomachs and small intestines were frozen in liquid nitrogen for DIOS-MS and –MSI analyses. Faeces were collected directly from the colon lumen for excretion data. Frozen tissue was stored at −80 °C until required for MS analysis.

### ACF and tumour scoring

Excised colons were straightened and cleaned to remove any faecal matter. They were then incised longitudinally and laid out on Hybond C membrane (NL1011; GE Healthcare) for fixation in 10% formalin for 24 h prior to being transferred to 70% ethanol. The samples were then stained with 0.2 g methylene blue in 100 ml saline for two to three minutes. The stained colons were viewed with a dissecting microscope, Olympus SZ51, under 400X magnification. ACF were identified as crypts that had increased size, elevated appearance from the surrounding mucosa, and with a slit-like shape of the luminal opening as reported by Di Gregario *et al*.^58^. Tumours were distinguished from large ACFs as they had no definite crypt openings on the surface and were larger in size.

### Measurement of apoptosis and cell proliferation

Following ACF and tumour scoring with methylene blue two to three mm slices of distal colon tissue on Hybond C were cut using a scalpel under the dissecting microscope so that areas of ACF and tumours were avoided. These sections underwent standard processing and paraffin-embedding before a microtome was used to cut (4–5 μm) sections which were placed on plain glass slides for haematoxylin staining or a Superfrost® Plus slides for Ki67 staining and air dried.

Haematoxylin staining was performed using standard techniques utilising Harris Haemotoxylin with acetic acid for 2 mins and dipped in acid ethanol and ammonia water for blueing and then mounted using DPX. A light microscope was used (Motic BA300, 400× magnification) to identify and count the number of apoptotic cell per crypt as described in Hu *et al*.^57^ and Le Leu *et al*.^59^.

The proliferative activity of colon epithelial cells was measured using immunohistochemical staining with a monoclonal antibody to nuclear proliferating antigen Ki-67. Sections were deparaffinised and rehydrated before antigen retrieval was performed by placing slides at 100 °C in 0.1 M citrate buffer for 40 min. The Ultra Streptavidin (USA) HRP Detection Kit Multi-Species, 3,3′-diaminobenzamine (DAB) substrate system (Covance Sig 32256, Biolegend, San Diego, CA, USA) was used to aid staining and detection of Ki67. After blocking sections were incubated overnight with a 1/200 dilution of Ki-67Ab (Abcam, Victoria, Australia: ab16667) and detection was carried out as per the manufacturer’s instruction. Slides were counterstained with Harris haematoxylin for 1 min, air dried and mounted using DPX. A light microscope (Motic BA300) under 400X magnification was used to observe and count Ki67 positive cells. Ten random crypts were chosen and counted as two halves. The crypt height and the number of proliferating cells were counted for each half and then a proliferation index was determined

### Surface fabrication of pSi

DIOS surfaces were fabricated using light assisted anodic etching of silicon wafers as described^5,9^ followed by ozone oxidation. Etched pSi surfaces were functionalised using neat silane (F_13_) for 60 min at 90 °C according to previously published methods^11,60^.

### Tissue preparation and DIOS-MSI

Stomach, duodenum, jejunum, ileum where mounted onto frozen stubs using optimum cutting temperature compound (OCT; Tissue-Tek®) while ensuring no OCT contaminated the tissue section to be imaged. Gastrointestinal tissue was cryo-sectioned to 12 μm thick at −20 °C and liver was cryo-sectioned to the same thickness at −14 °C (Leica 1800 Cryostat, Leica Microsystems). The fundus portion of the stomach was sectioned down until the frozen gut content was exposed, to evaluate metabolism within the stomach as well as absorption into the mucosa. Small intestinal segments and livers were cryo-sectioned until a clean surface was created for effective serial sectioning. For each tissue section used for DIOS-MSI a serial section was cut for H & E. Sections for DIOS-MSI were thaw mounted onto the DIOS surface and placed in a desiccator for 30 min to allow surface imprinting, followed by digital scanning (Epson V700). The residual tissue was then removed from the DIOS surface with a gentle stream of 25 °C Milli-Q water from a pipette prior to MSI. Sections for H & E were mounted onto polyethyleneimine (PEI) polymer coated slides^61^, allowed to air dry in a desiccator and stained using a standard protocol. To separate acid condensation products of 6Br from xenobiotic metabolites, 0.1 mg/g, 0.05 mg/g and 0.01 mg/g (6Br per mg control mice weight) was homogenised with control mouse stomach tissue, and heated at 37 °C with 1 mL of lysis buffer (Tris-Cl 100 mM, NaCl 200 nM, SDS 10% w/v, EDTA 5 mM and Proteinase K 100ug/mL in H_2_O) for 1 h in microfuge tubes. The homogenised tissue was then frozen in liquid nitrogen, cryo-sectioned as previously described and imprinted on pSi.

### DIOS-MSI analysis

DIOS-MSI analysis was performed on an ultrafleXtreme™ MALDI-TOF/TOF mass spectrometer (Bruker-Daltonics) equipped with a smartbeam-II™ 2 kHz pulsed laser operating at 2 kHz repetition rate in reflectron positive and reflectron negative ion mode. Spectra were collected in the 20-1500 Da range at a spatial resolution of 60 µm. Laser diameter was set to medium, corresponding to ~50 µm. A total of 500 laser shots were acquired at each sampling point/pixel. The imaging area was defined using flexImaging 3.4 (Bruker-Daltonics) and data acquired using flexControl 3.3 (Bruker-Daltonics). External quadratic calibration was achieved using caesium iodide (CsI) mixed with 2-[(2E)-3-(4-tert-Butylphenyl)-2-methylprop-2-enylidene]malononitrile (DCTB) in a 1:1 ratio of 5 µL (10 mg/mL CsI) to 5 µl (10 mg/mL DCTB)^62^. Calibration was based on CsI adducts including: Cs 132.90490, CsI 392.71483, (Cs)_2_ 652.52475, (Cs)_3_ 912.33468, (Cs)_4_ 1172.14460, and (Cs)_5_ 1431.95453 *m/z*. Ion intensity maps were generated using SCiLS Lab (version 2.02.5366, SCiLS GmbH, Bremen, Germany) and flexImaging after baseline subtraction (TopHat), data reduction (resampled TIC preserving) and peak selection.

### Statistical analysis

Summed processed spectra, with reference to the serial H & E section, were analysed by pLSA to establish likeness to tissue regions and define the ‘spectral environment’ mono and di-brominated signatures were detected in. Peak clusters that fit the typical mono and di-brominated spectral pattern were further analysed by spatial correlation analysis to find co-localised brominated signatures. Spectral patterns of interest were referenced back to the homogenised control spiked with 0.1, 0.05, and 0.01 mg/g 6Br and TP standard.

The statistical analysis for the animal model was performed using GraphPad (Prism 6). One way ANOVA was performed to compare the treatment groups to the control group. A Bonferroni multiple comparison test was applied to see the significant differences between the control and the groups. P values ≤ 0.05 were considered to be statistically significant.

### Gas chromatography-mass spectrometry (GC-MS)

To capture all potential brominated metabolites, confirm DIOS-MSI data and annotate the effective description of the tissue regions developed using pLSA analysis, the remaining tissue of the imaged section was rinsed in Milli-Q water and extracted using a modified lipid extraction procedure^63^. Briefly, tissue was macerated in a chilled solvent solution of chloroform and methanol (2:1) to a volume 20 times that of the weight of tissue (w/v). After extraction on an orbital shaker for 20 min, homogenates were vacuum filtered, and washed using 0.9% w/v sodium chloride (NaCl) solution until phase separation occurred. The chloroform fraction was filtered through a phase separation medium (silicone phase separation paper; GE), dried under vacuum and resuspended in hexane at a concentration of 50 mg/mL for LC-MS injection or 10 mg/mL for GC-MS injection.

GC-MS analysis was achieved on a Hewlett Packard 6890 Gas Chromatography unit coupled to a HP 5973 Mass Selective Detector, with helium as the carrier gas. Fatty acyls and brominated indoles were separated on a HP-5MS capillary column (Crosslinked 5% PH ME Siloxane; Agilent, model 19091S-433) with dimensions 30 m x 0.25 mm and 0.25 μm film thickness. Samples were run in split mode.

Lipids were extracted as above for GC-MS but resuspended in hexane at a concentration of 10 mg/mL for LC-MS injection or capillary spotting on to TLC plates, as previously described^11^. Lipid samples not immediately used were stored suspended in hexane at −80 °C.

### LC-MS analysis of lipid classes and brominated indoles

Total lipid extracts were analysed using a modified lipid class HPLC-MS method^64^ with the aim of capturing all possible lipid groups rather than full chromatographic separation of each lipid class, as lipid species are too numerous for effective separation within a single run without highly specialised double column arrangements. Chromatography was performed on a Phenomenex Luna C18 (2) HPLC column (100 Å, 250 × 4.6 mm) with isopropanol (IPA) containing 0.005% trifluoroacetic acid (v/v; TFA) and methanol containing 0.005% TFA (v/v). Samples were loaded as a 2 μL injection volume and separated using a flow rate of 0.75 mL/min for 53 min. Separation of major lipid classes was achieved by a gradient of 10% IPA held for 30 min, changed to 25% IPA for 10 min, changed to 95% IPA 5 min, held at 95% IPA for 2 min, changed to 10% IPA for 3 min and held at 10% IPA for 3 min. Lipids were analysed on a Agilent 1260 Infinity HPLC system coupled to a 6120 Quad mass spectrometer, with diode array detector (G4212B), binary pump (G4220A) and auto-sampler (G4226A) using ESI. Lipid matches to DIOS and Ag-DIOS was performed using the extract ion function in Agilent Chemstation software.

### HP-TLC-MALDI analysis of lipid classes

Lipid classes were determined on TLC-MALDI plates as previously reported^11^. Briefly, a total of 1 μL of the lipid extract was spotted onto specifically designed HP-TLC-MALDI plates (aluminium backed silica gel 60 F_254_, 200 μm thick, 50 × 75 mm; Merck) and separated in a solvent gradient of chloroform, ethanol, water and triethylamine (35:35:7:35, v/v/v/v). The prepared TLC plate was scanned and coated with ten layers of 100 mg/mL 2,5-dihydroxybenzoic acid (2,5-DHB in 70% v/v methanol) using an iMatrixSpray robot^65^. MALDI-MSI was conducted on an ultrafleXtreme MALDI-ToF/ToF instrument (Bruker) operating in reflectron positive ion mode at 500 shots per position at a spatial resolution of 250 µm across each TLC lane. External calibration used 2,5-DHB peaks and peptide calibration standard II, spotted directly onto the TLC plate prior to matrix deposition. MS/MS spectra were acquired from areas of high ion. These data were also used to produce figures for the supporting information of a preceding publication^11^.

## Supplementary information

Supplementary Information
