# Supplementary material for: Mapping insoluble indole metabolites in the gastrointestinal environment of a murine colorectal cancer model using desorption/ionisation on porous silicon imaging
Source: Sci Rep. 2019 Aug 26;9:12342. doi: 10.1038/s41598-019-48533-2 (PMC6710270; doi:10.1038/s41598-019-48533-2)
Supplement: Supplementary file 1 — Supplementary Information [file 41598_2019_48533_MOESM1_ESM.docx]

**Mapping insoluble indole metabolites in the gastrointestinal environment of a murine colorectal cancer model using desorption/ionisation on porous silicon imaging**

David Andre Rudd, Kirsten Benkendorff, Charndeep Chahal, Taryn Guinan, Ove Johan Ragnar Gustafsson, Babak Esmaeelian, Hanna Krysinska, Lisa Pogson, Nicolas Hans Voelcker and Catherine Anne Abbott

Supplementary Information

Our study demonstrates a novel approach to *in vivo* drug efficacy models by further utilising the tissue for mass spectrometry imaging to identify the drug metabolites present throughout the gastrointestinal tract. In order to detect the low abundant drug metabolites and discriminate them from endogenous primary metabolites, the detection method must be suitable in capturing all potential drug compounds generated *in vivo*, which in this case includes the highly insoluble 6,6’-dibromoindigo and 6,6’-dibromoindirubin (Gustafsson et al., 2017). The method must also be able to discriminate drug metabolites from primary metabolites that will be present in a MSI data set, so partial annotation of tissue extracts is required to compare controls to orally administered treatment groups. This supplementary information contains data demonstrating the effectiveness of DIOS-MS to detect drug metabolites, the annotation of tissue extracts and comparison to DIOS-MSI and other supporting information for therapeutic claims around 6 bromoisatin (6Br) and the natural extract (NE) it originates from.


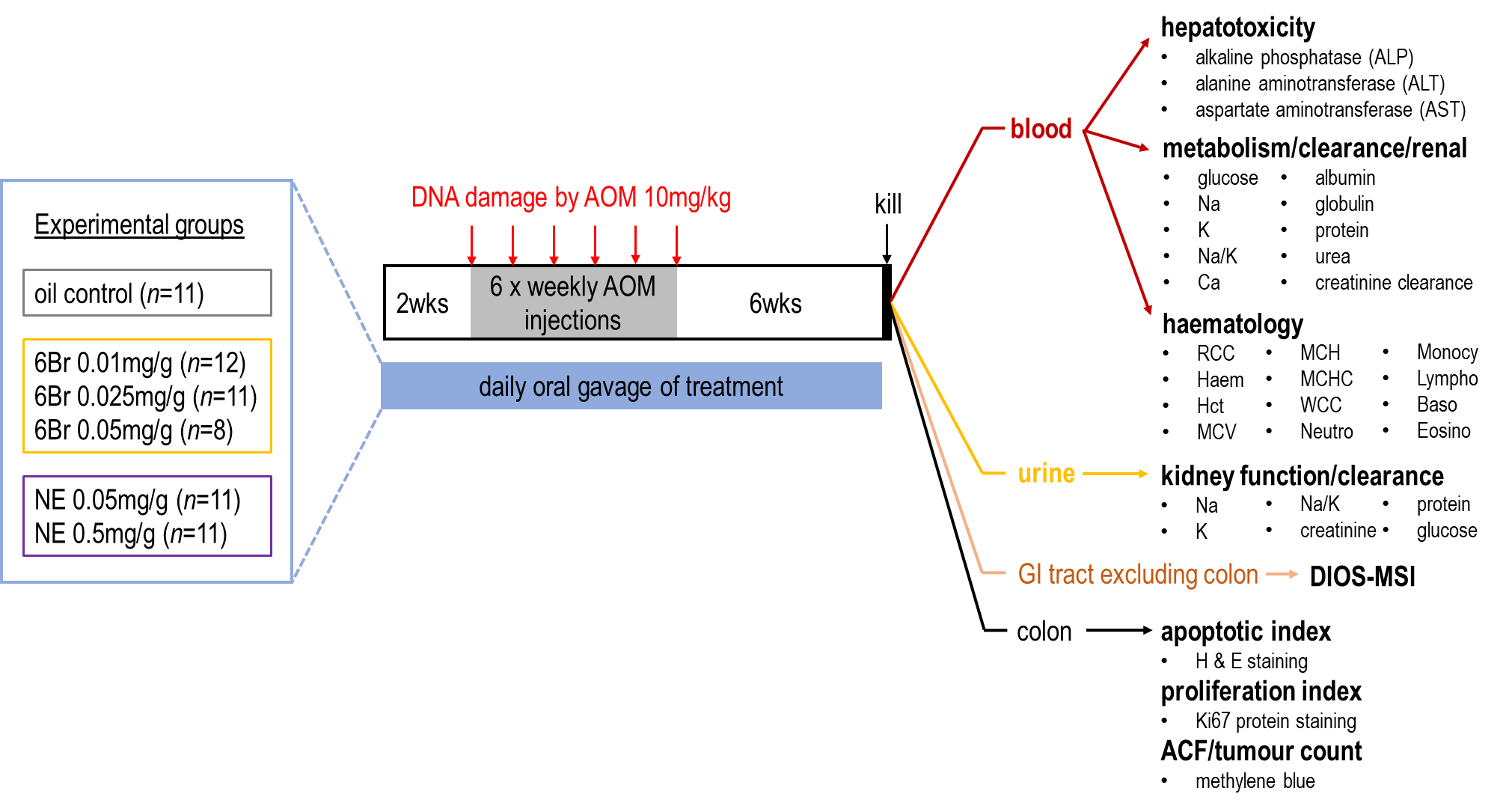


**Supplementary Figure 1**. Experimental workflow to measure the efficacy of 6Br and NE in preventing ACF and tumour formation in a genotoxin colorectal cancer mouse model. Haematology measures include: RCC, red blood cell count; Haem, haemoglobin; Hct, haematocrit; MCV, mean corpuscular volume; MCH, mean corpuscular haemoglobin; MCHC, mean corpuscular haemoglobin concentration; WCC, white blood cell count; Neutro, neutrophils; Monocy, monocytes; Lympho, lymphocytes; Baso, basophils; Eosin, eosinophils.

**
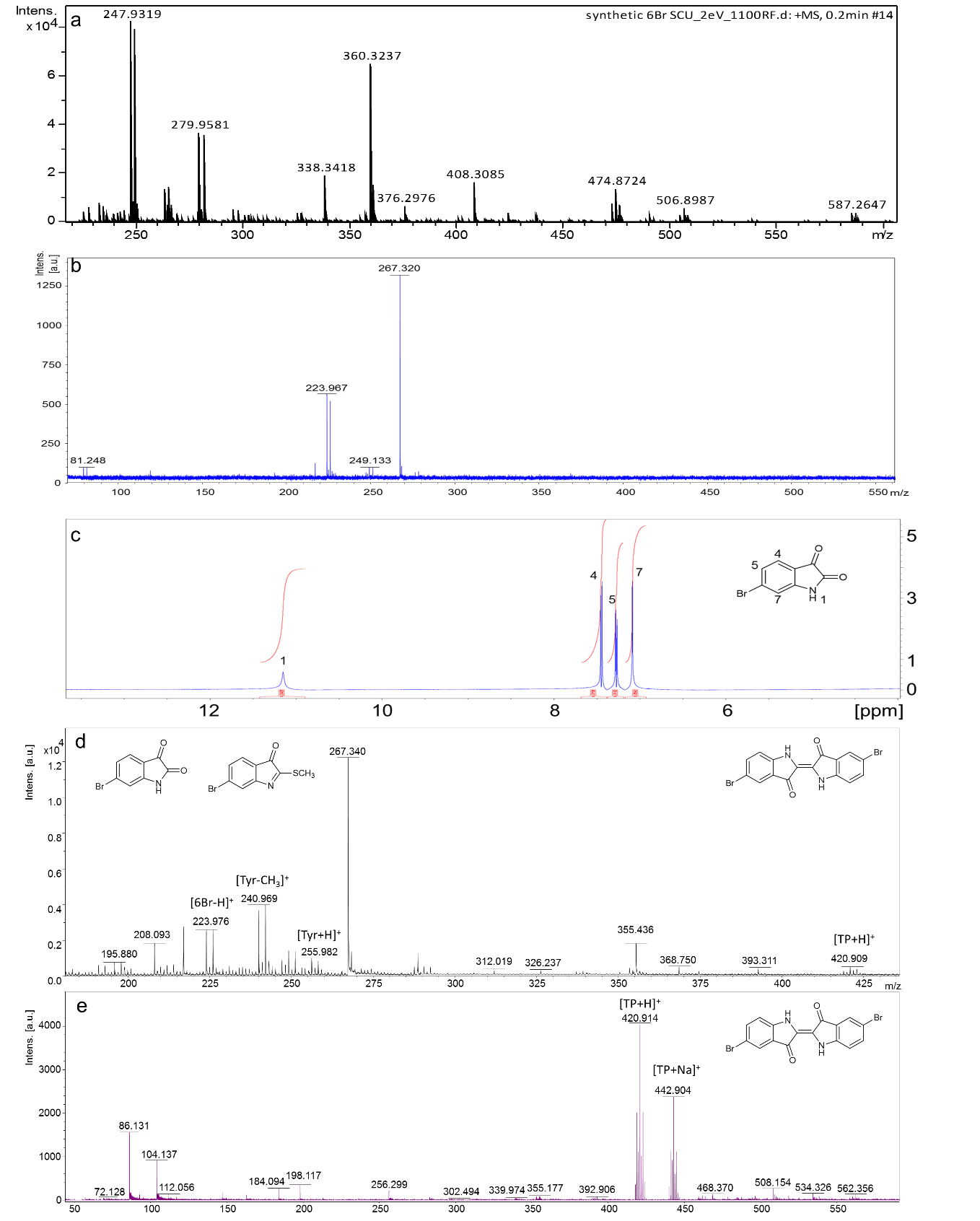
**

**Supplementary Figure 2**. 6Br and NE when analysed with different techniques (a) ESI-ultra high resolution-quadrupole time of flight MS (ESI-UHR-QqTOF) mass spectrum of pure synthetic 6Br, (b) 6Br analysed with DIOS-MS and (c) corresponding proton 1 H-NMR of synthetic standard in deuterated methanol. For the analysis of 6Br, proton impact in the ion source can lead to hydrogen extraction and result in [M-H]+ m/z 223.9, 225.9, as seen in panel (b). (d) DIOS-MS of NE, prior to oral administration showing brominated ions for 6Br, Tyr and TP. (e) DIOS-MS of Tyrian purple standard homogenised in stomach control for comparison to TP formation in the stomach from NE.


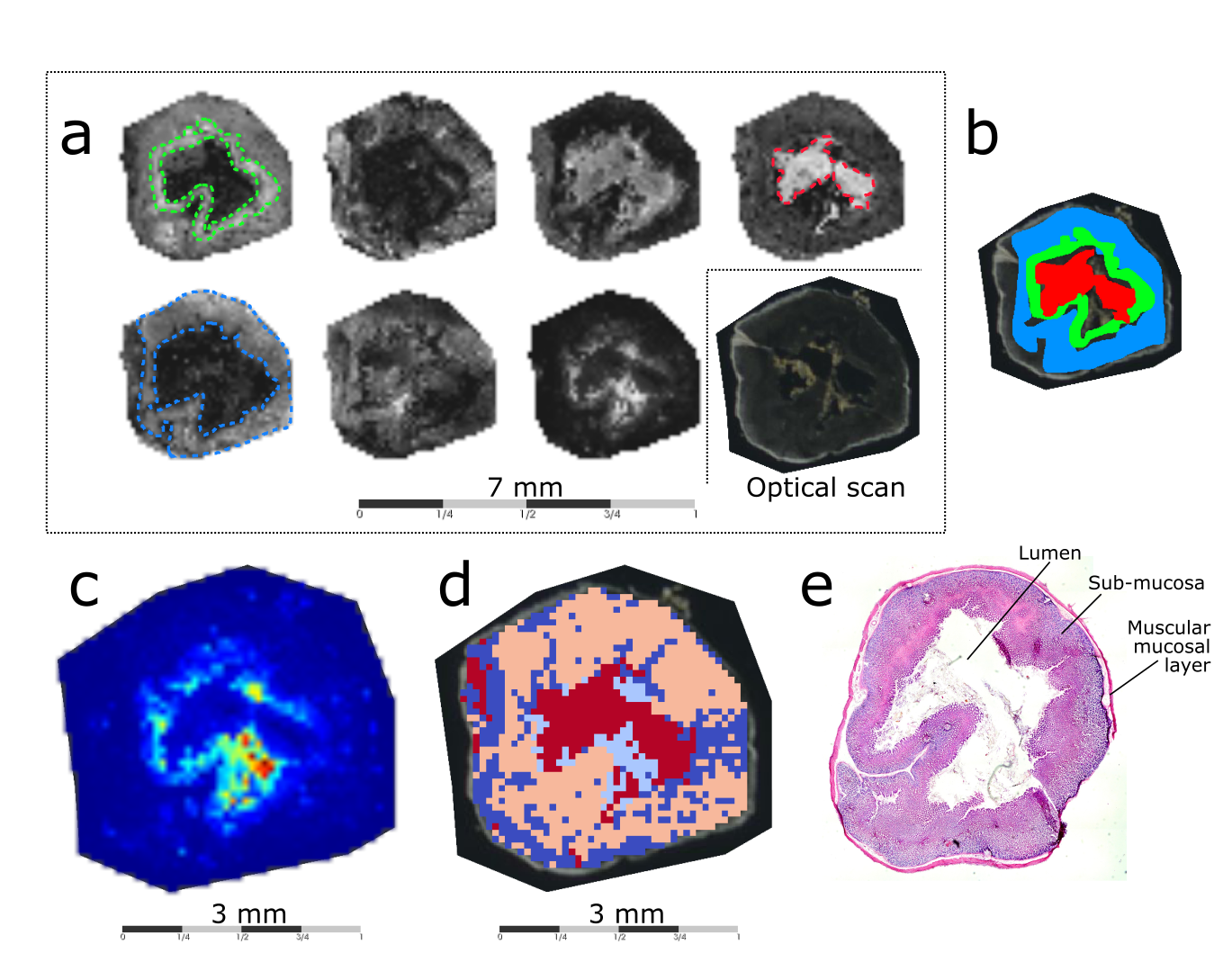


**Supplementary Figure 3**. Comparison of probabilistic latent semantic analysis (pLSA) to stomach tissue histology. Panel (a) shows a seven component pLSA (random initialization of total ion count [TIC] normalised data with an interval width of ± 0.1 Da) using aligned peak intervals from the tissue-only area of the MSI data (manually defined). The bottom right image in (a) is an optical scan of the tissue section on the DIOS substrate. Tissue regions manually defined in (a) - user coloured dotted lines drawn on the pLSA heat maps is overlayed with the optical tissue section scan in (b). An example TIC normalised ion intensity map for the mucosa is provided in (c) for m/z 279.259 ± 0.1 Da. A k-means (k =4) cluster analysis (cosine distance) in panel (d), and histological stain of a consecutive section in €, further support the molecular-definition of these tissue compartments. Tissue compartment annotations are provided in (e), and panel (c) and (d). Analysis was performed in SCiLS 2015b (SCiLS GmbH).


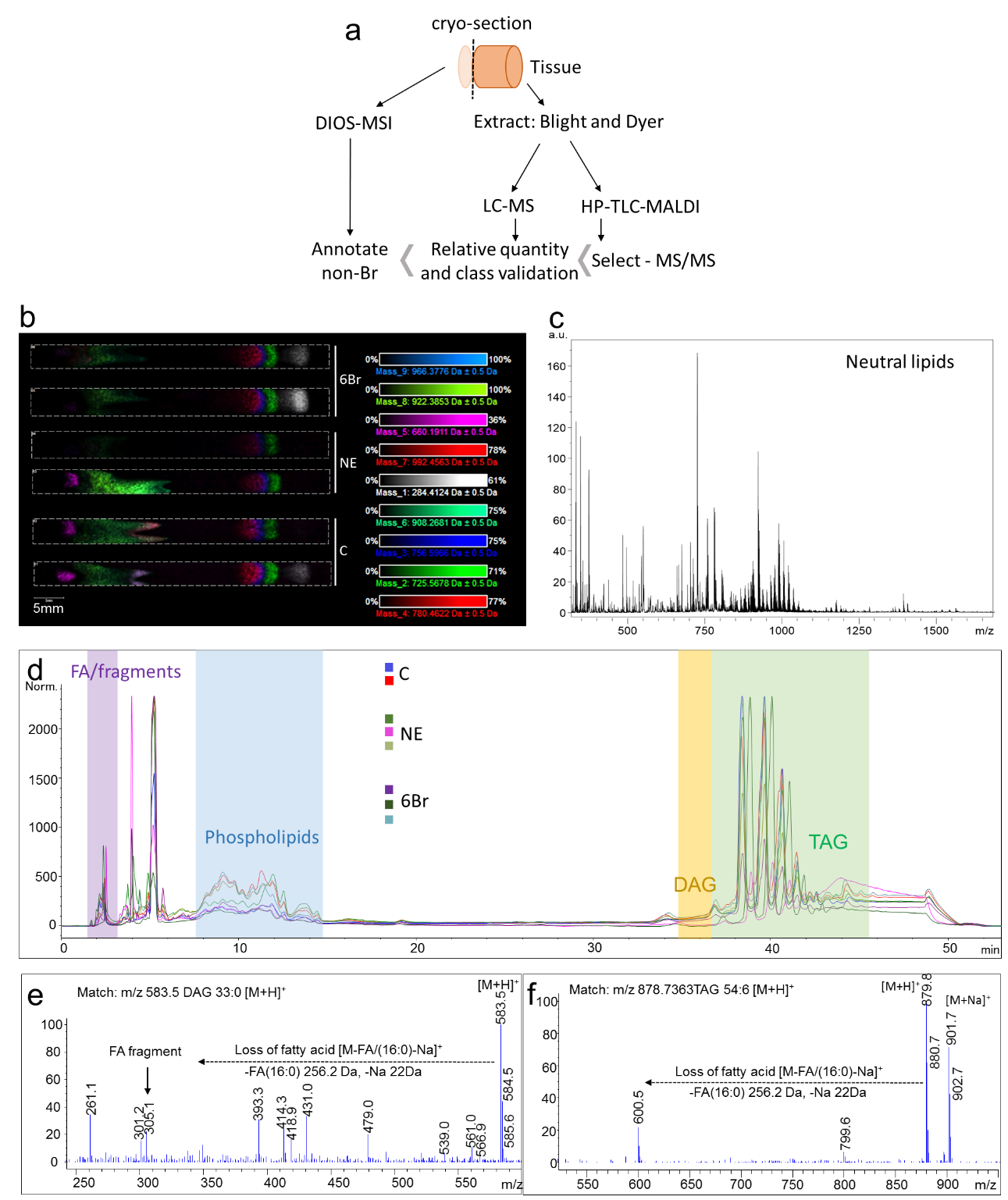


**Supplementary Figure 4**. Annotating ions in DIOS-MSI maps from neutral lipids by extraction of residual tissue and running LC-MS and HP-TLC-MALDI. (a) Workflow for lipid analysis. (b) HP-TLC-MALDI plate MSI image with selected lipid spots separated using a solvent gradient of chloroform, ethanol, water and triethylamine (35:35:7:35, v/v/v/v). (c) Summed spectrum for neutral lipids from HP-TLC-MALDI data used to confirm the absence of drug metabolite signals in the control samples. (d) Overlaid LC-MS chromatograms of neutral lipid extracts used to investigate how LC-MS compares to DIOS-MS in capturing brominated drug metabolites and differentiating them from lipid signatures. LC-ESI-MS spectra can be used to annotate lipid groups including diacylglycerides (e) and triacylglycerides (f) in combination with MALDI-TLC. Labelling: FA/fragments, fatty acids or fragments; PL, phospholipids; PC, phosphocholines; GL/DAG, unannotated glycerolipids and diacylglycerides; TG, triacylglycerides.


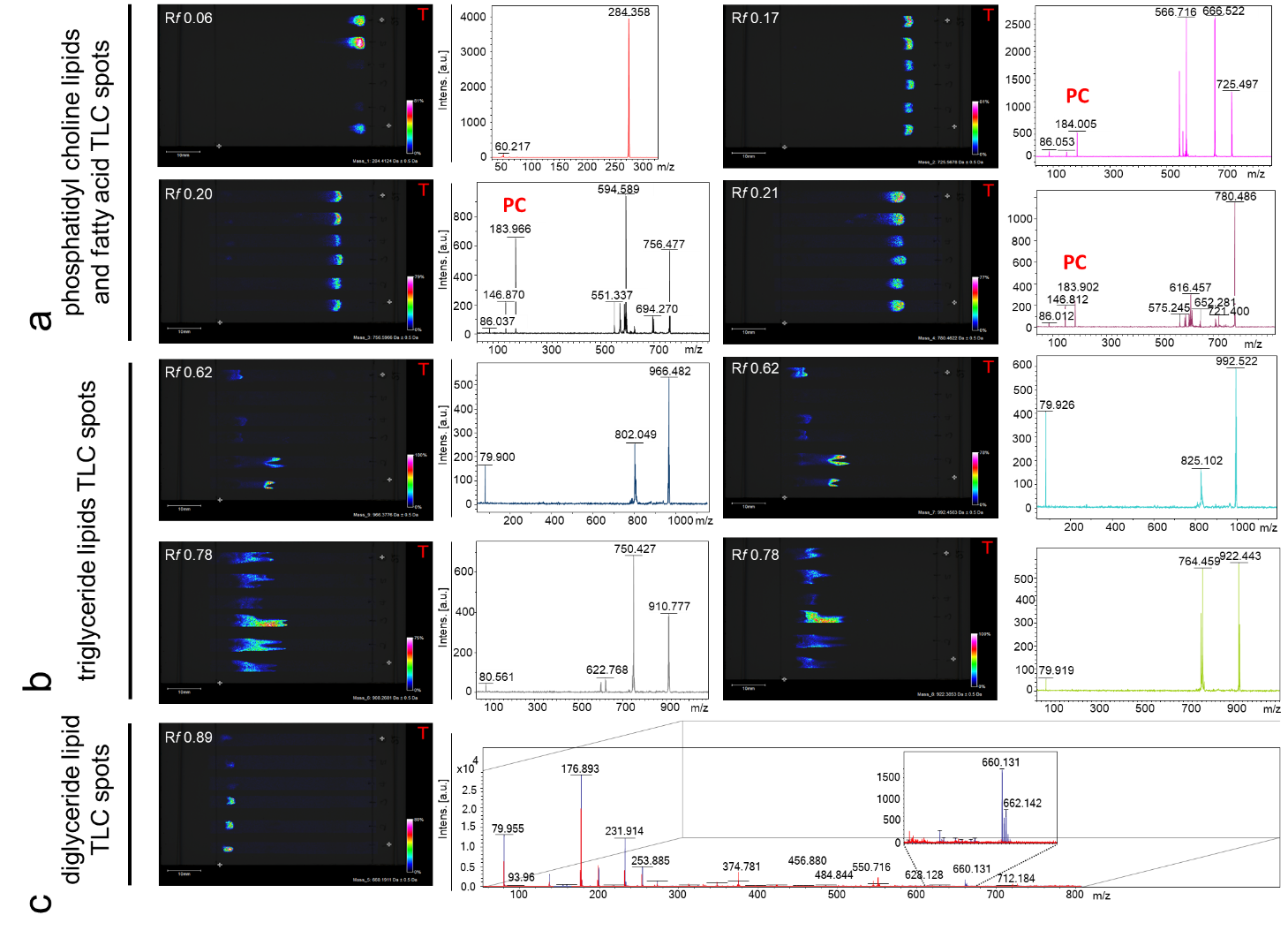


**Supplementary Figure 5.** Selected MS/MS from HP-TLC-MALDI spots for deconvolution of neutral lipid ions from DIOS-MSI data to compliment LC-ESI-MS data of the same extract. PC, phosphatidylcholine based on MS/MS with indicative head-group ion. Panel (a) shows phosphatidyl choline lipid TLC spots with corresponding MS/MS spectra. Panel (b) shows triglyceride lipid TLC spots with corresponding MS/MS spectra. Panel (c) shows diglyceride lipid TLC spots with corresponding MS/MS spectra.


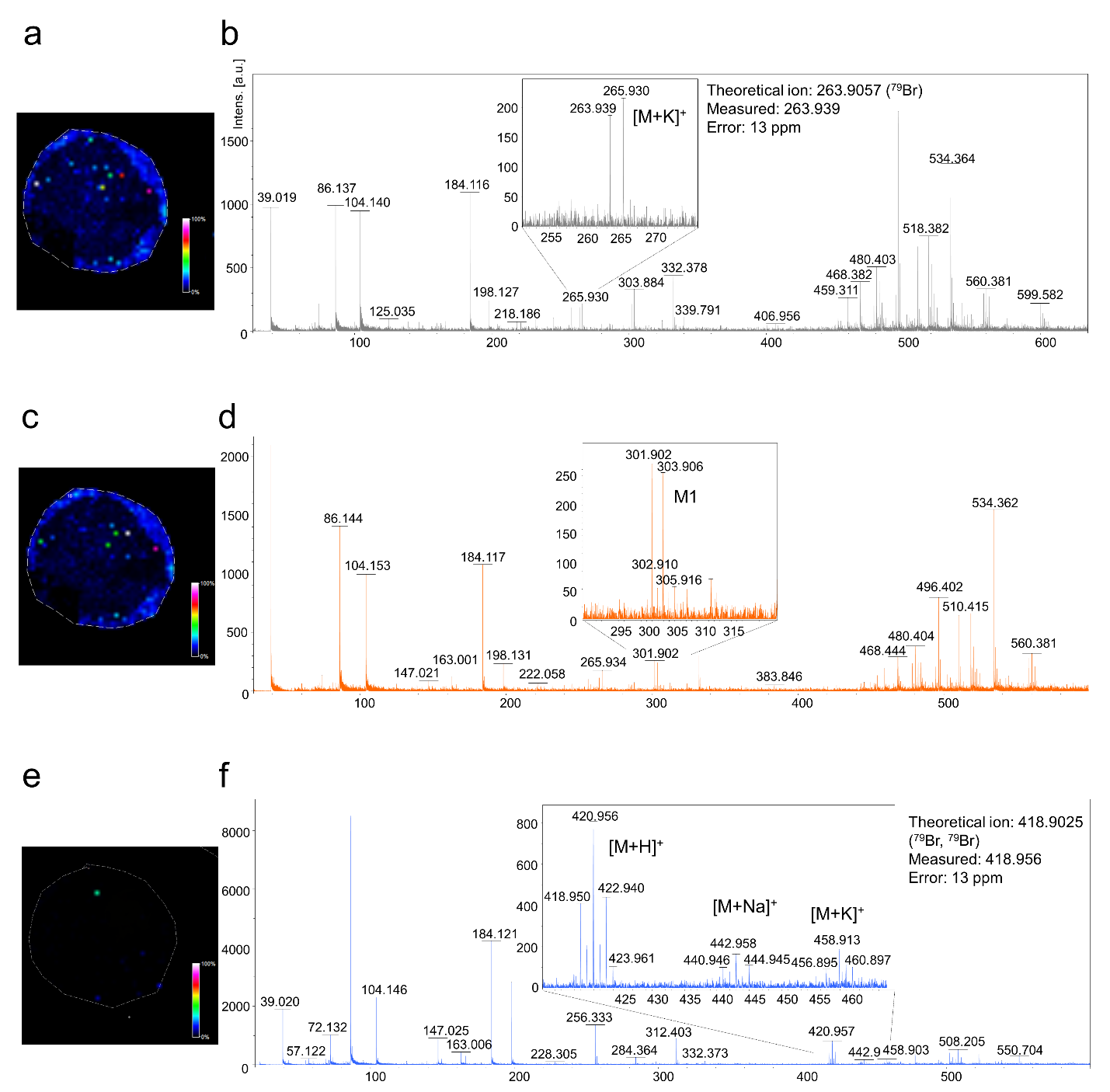


**Supplementary Figure 6**. DIOS-MSI of homogenised stomach tissue of control mice with 6Br for positive confirmation of Br ions in DIOS-MSI. (a) homogenized stomach with 6Br injected and imaged via DIOS-MS, with (b) corresponding spectra from highest intensity pixel. (c) homogenised stomach tissue from 6Br group showing metabolite, M1, with (d) corresponding spectra. (e) homogenised stomach tissue with injected synthetic TP (6,6’-dibromoindigo), with (f) corresponding spectra.


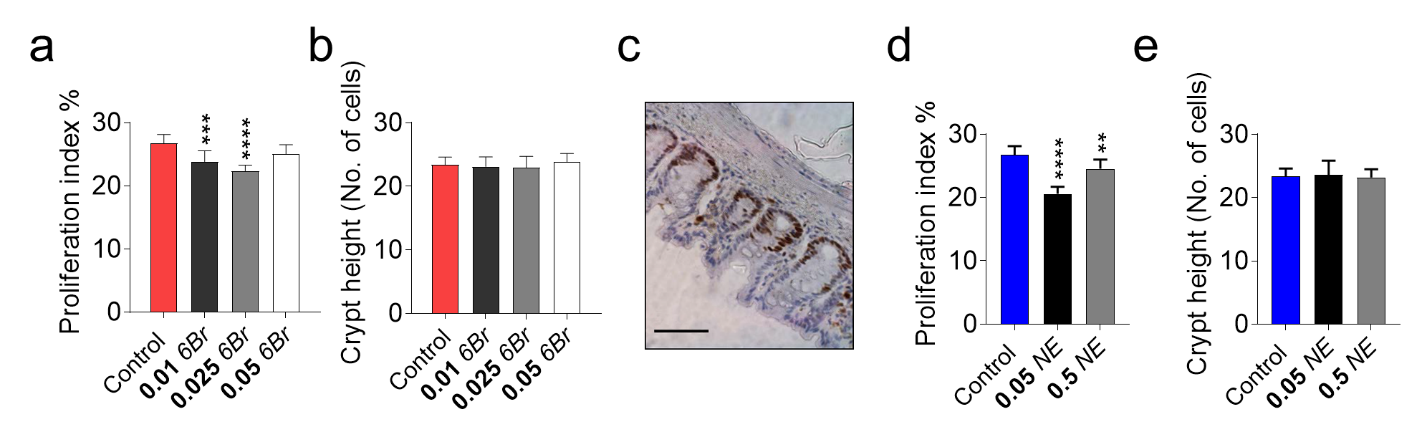


**Supplementary Figure 7.** 6-Bromoisatin (6-Br, mg/g) and NE (mg/g) reduce proliferation index but have no effect on average crypt height. Proliferation index (%) in control mice compared to mice treated with 6Br (a) and NE (d). (c) representative image of Ki67 staining used to detect proliferating cells, scale bar 50 µm. Crypt height in distal colon from control mice compared to mice treated with 6Br (b) and NE (e). All treatment groups are compared to control mice by ANOVA. P≤0.01(**), P≤ 0.005 (***) and P≤ 0.0001 (****).


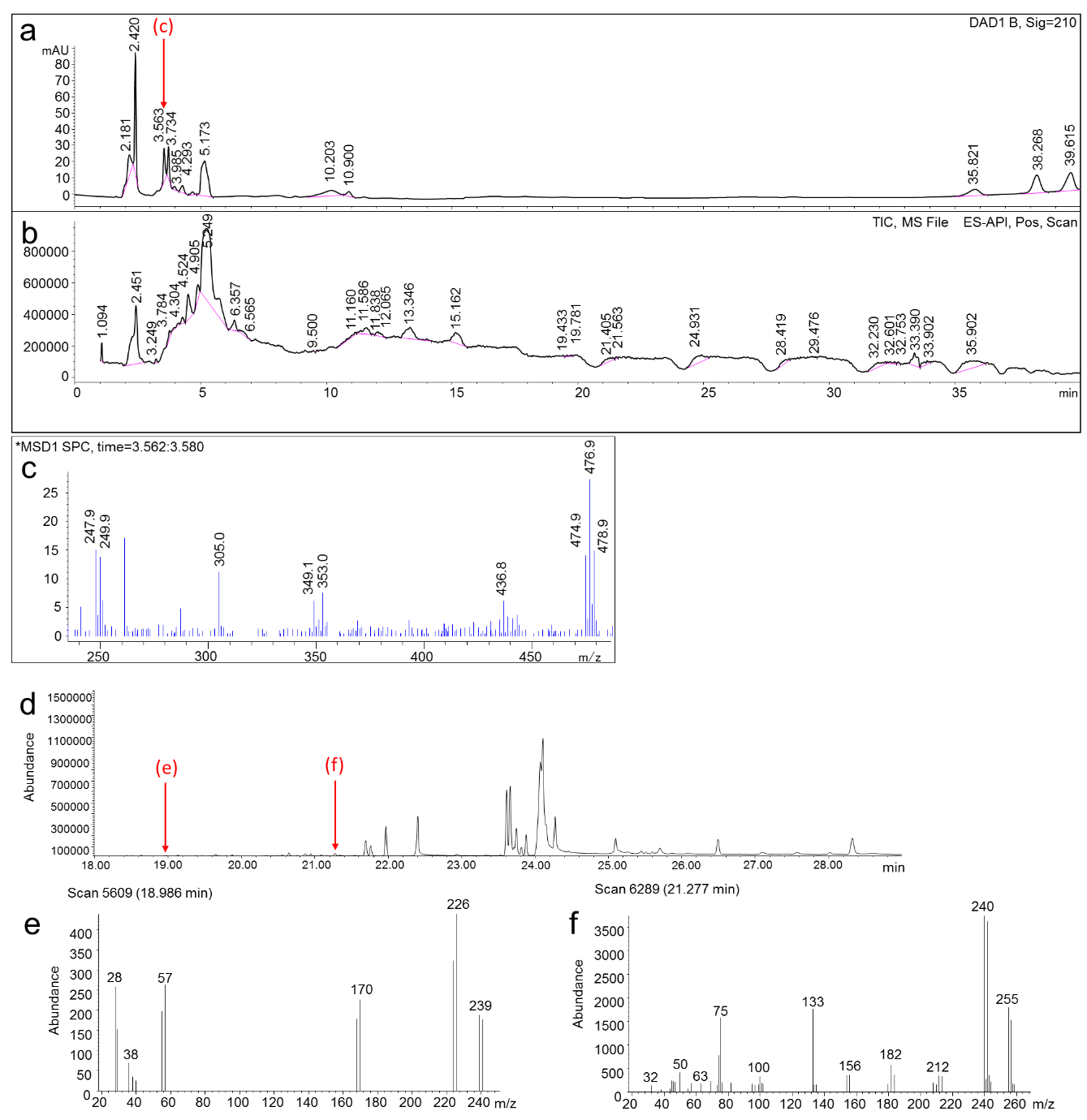


**Supplementary Figure 8.** LC-MS and GC-MS of the stomach extracts of mice orally administered 0.5 mg/g NE showing the brominated indole metabolites detected from the same tissue used for DIOS-MSI. HPLC chromatogram from the diode array detector (DAD) at 210 nm (a) and total ion current (TIC) of ESI-MS in positive mode (b) of the chloroform fraction of a methanol:chloroform partition from stomach extracts showing lipophilic primary metabolites including a brominated indole metabolite dimer (c). (d) GC-MS chromatogram showing a further two mono-brominated ion from the stomach extract including tentative metabolite [6Br+CH3] + (e), and tyrindoleninone [M]+• (f).


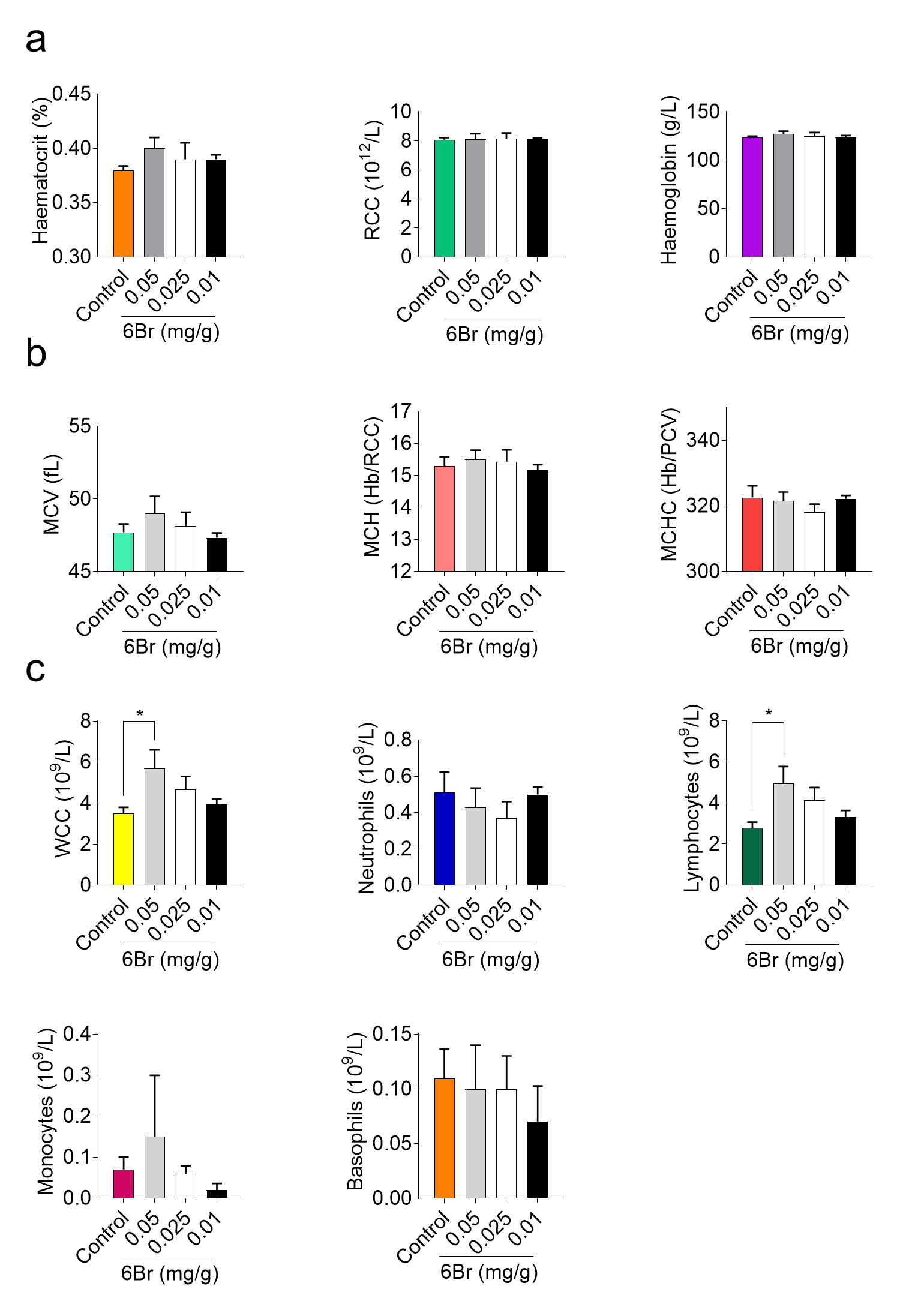


**Supplementary Figure 9**. Haematological counts and cell morphology for mice orally administered 6Br or oil control, collected using cardiac puncture. General blood parameters (a), red blood cell indices (b), white blood cell count and proportion (c), assessed by an independent pathology clinic (Gribbles Pathology). RCC, red blood cell count; MCV, mean corpuscular volume; MCH, mean corpuscular haemoglobin (Hg); MCHC, mean corpuscular haemoglobin concentration; WCC, white blood cell count. All treatment groups are compared to control mice by ANOVA. P≤0.05(*)


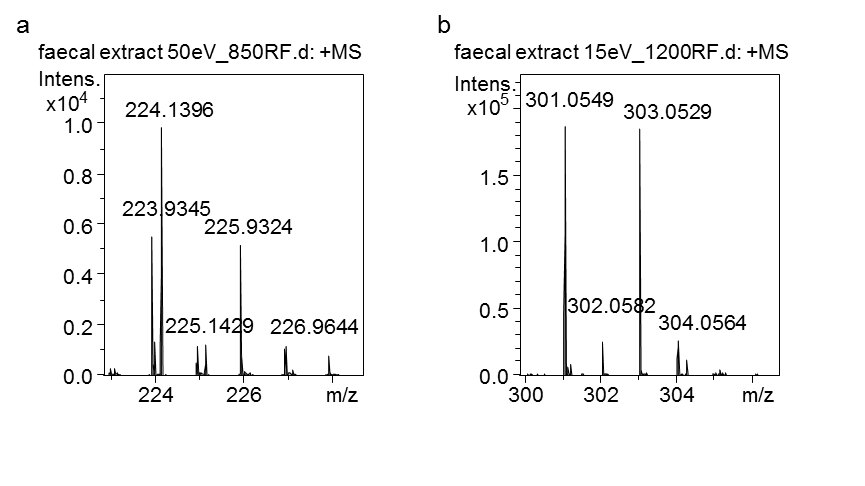


**Supplementary Figure 10**. ESI-ultra high resolution-quadrupole time-of-flight (ESI-UHR-QqTOF) MS of faecal extract from mice orally administered 0.05 mg/g 6Br, showing (a), 6Br and (b), metabolite **M3** (Fig. 4a).

**Supplementary Figure 11**. Haematological counts and cell morphology for mice orally administered NE or oil control, collected using cardiac puncture. General blood parameters (a), red blood cell indices (b), white blood cell count and proportion (c), assessed by an independent pathology clinic (Gribbles Pathology). RCC, red blood cell count; MCV, mean corpuscular volume; MCH, mean corpuscular haemoglobin (Hg); MCHC, mean corpuscular haemoglobin concentration; WCC, white blood cell count
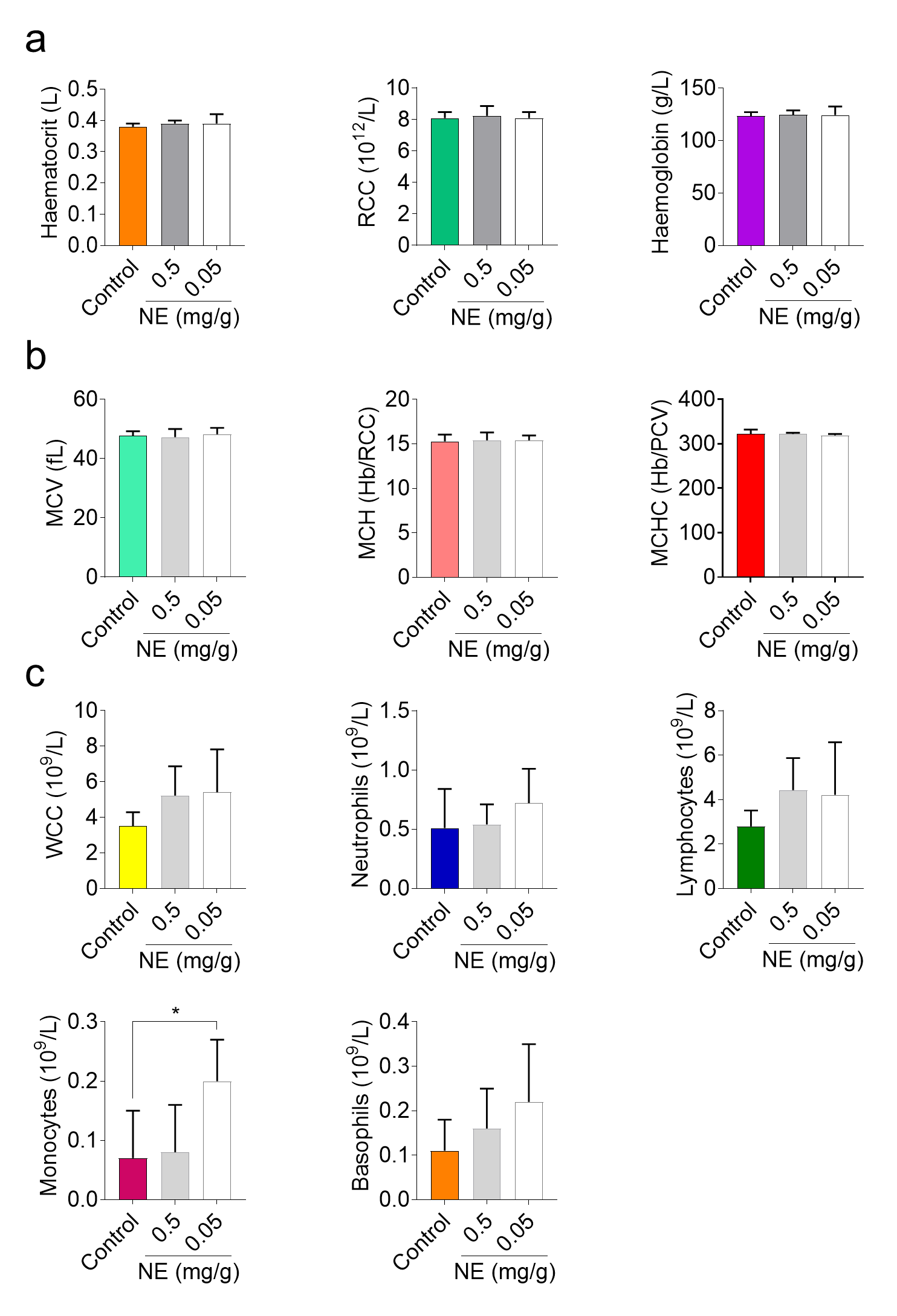
. All treatment groups are compared to control mice by ANOVA. P≤0.05(*)

**References**

GUSTAFSSON, O. J. R., GUINAN, T. M., RUDD, D., KOBUS, H., BENKENDORFF, K. & VOELCKER, N. H. 2017. Metabolite mapping by consecutive nanostructure and silver-assisted mass spectrometry imaging on tissue sections. *Rapid Communications in Mass Spectrometry,* 31**,** 991-1000.
